# An optical probe for detecting chondrocyte apoptosis in response to mechanical injury

**DOI:** 10.1038/s41598-017-10653-y

**Published:** 2017-09-07

**Authors:** Yihui Huang, Jun Zhou, Amirhossein Hakamivala, Jinglei Wu, Yi Hong, Joseph Borrelli, Liping Tang

**Affiliations:** 1 Department of Bioengineering, University of Texas at Arlington, P.O. Box 19138, Arlington, TX 76019 USA; 2 Texas Health Physicians Group, Texas Health Arlington Memorial Hospital, Arlington, TX 76012 USA; 3 Department of Biomedical Science and Environmental Biology, Kaohsiung Medical University, Kaohsiung, 807 Taiwan

## Abstract

Cartilage injury induced by acute excessive contact stress is common and mostly affects young adult. Although early detection of cartilage injury may prevent serious and lifelong arthritic complications, early detection and treatment is not possible due to the lack of a reliable detection method. Since chondrocyte injury and subsequent cell death are the early signs of cartilage injury, it is likely that cartilage cell apoptosis can be used to predict the extent of injury. To test this hypothesis, a near infrared probe was fabricated to have high affinity to apoptotic cells. *In vitro* tests show that this apoptosis probe has low toxicity, high specificity, and high affinity to apoptotic cells. In addition, there is a positive relationship between apoptotic cell numbers and fluorescence intensities. Using a mouse xiphoid injury model, we found significant accumulation of the apoptosis probes at the injured xiphoid cartilage site. There was also a positive correlation between probe accumulation and the number of apoptotic chondrocytes within the injured xiphoid cartilage, which was confirmed by TUNEL assay. The results support that the apoptosis probes may serve as a powerful tool to monitor the extent of mechanical force-induced cartilage injury *in vivo*.

## Introduction

It is estimated that more than 27 million adults in the United States suffer from osteoarthritis, approximately 12% of these individuals have a type of osteoarthritis that develops due to a mechanical force-induced cartilage injury (also called as post-traumatic osteoarthritis)^1, 2^. Patients with cartilage injury commonly suffer from joint pain and stiffness, reduced mobility and function, poor quality of life, and increased lifetime medical costs^3–6^. Unfortunately, injured cartilage may worsen and remain asymptomatic for years. When symptoms arise, the disease may have already reached a later, more complicated stage that is compounded by limited treatment options.

The diagnosis of injured cartilage generally begins with a detailed history, a physical examination, and plain radiographs of the joint. Certain laboratory tests (e.g., synovial fluid tests, computed tomography scans, magnetic resonance imaging) may be useful to confirm the presence of injured cartilage^3, 7^. Magnetic resonance images provide a three-dimensional view of cartilage that may elucidate cartilage irregularities^8^. Since these structural changes can only be appreciated during the intermediate or late stages of cartilage injury, these conventional methods cannot be used to detect early changes within the injured cartilage. It is generally believed that the early diagnosis and prevention of cartilage injury will improve outcomes while reducing disability and associated costs^9^. Therefore, there is an urgent need for a method that will detect cartilage injury at its early stage.

Noninvasive near-infrared (NIR) fluorescent imaging technology has recently been used for the detection of various diseases and physiological conditions, including cancer, arthritis, infection, and cardiovascular disease^10–14^. NIR light can penetrate deeper into tissues (up to 10 cm depending on the tissue types) than visible light because biological tissues absorb less NIR light^15^. In addition, due to less autofluorescence with the use of NIR imaging, a higher signal-to-background acquisition ratio is possible^16^. To exploit the unique features of NIR light for *in vivo* imaging, several optical imaging probes have been developed to diagnose cartilage injury during its earliest stages. For example, NIR probes have been prepared to detect cathepsin and matrix metalloproteinases in synovial fluid, because higher levels of these components were found in patients with arthritis^17–19^. Hyaluronic acid–immobilized gold nanoprobes have been used for the *in vivo* monitoring of the high local production of reactive oxygen species and hyaluronidase in arthritic joints^20^. A folate-targeted NIR probe has been used *in vivo* to image rheumatoid arthritis in mice; namely, this is because the synovial membrane in mice with rheumatoid arthritis has been shown to accumulate large numbers of activated macrophages, which have upregulated folate receptors^21^. In general, these probes have been developed to detect inflammatory responses and products associated with inflammatory arthritis.

On the other hand, chondrocyte apoptosis is well recognized as one of the earliest responses to cartilage injury caused by mechanical trauma and is linked with injury severity^22–25^. Chondrocyte apoptosis has been associated with the production of reactive oxygen species, the shortage of growth factors, the release of glycosaminoglycan, and mechanical injury^22, 26^. After chondrocyte apoptosis, there is a loss of resident cells in the articular cartilage, which results in impaired extracellular matrix and potentially in the development of cartilage injury^27, 28^. Therefore, we believe that the detection of apoptotic chondrocytes soon after cartilage injury can be used to determine the extent of the cartilage injury that can possibly lead to early treatment and prevention.

Many groups of imaging probes have been developed for the detection of different stages of cell apoptosis. Annexin V^29^, synaptotagmin C2A domain^30^, and phosphatidylserine-binding peptides^31^ have been developed to recognize phosphatidylserine. It was later found that phosphatidylserine may also be present in the outer leaflets of non-apoptotic cells, including activated B and T lymphocytes and mast cells^32^. In addition, a class of apoptosis probes that are classified based on their ability to determine activated caspases have also been described^33, 34^. Despite their high specificity, caspase-based probes only recognize intracellular molecules and must be internalized for detection, which limits their use for *in vivo* imaging. A third group of apoptosis probes is a family of small molecules that includes N, N′-didansyl-L-cysteine and butyl-2-methyl-malonic acid^35, 36^. These compounds can selectively bind to transport membranes. They then accumulate in the cytoplasm of apoptotic cells following the cells’ permanent loss of their membrane potential, membrane acidification, and phospholipid scrambling^36^.

Another class of apoptosis probes is based on a monoclonal antibody that recognizes the La autoantigen, which is present only after loss of cell membrane integrity during the later stages of apoptosis^37, 38^. To improve the ability to detect cell apoptosis *in vivo* during the earliest stages, ApoPep-1—a six-amino-acid peptide (CQRPPR)—has been developed. This probe would target apoptotic cells by binding to histone H1, which is exposed on the surface of apoptotic cells^39, 40^. However, it is not clear whether the ApoPep-1 probe can be fabricated for the *in vivo* detection of injured cartilages.

In the current study, an apoptotic cell–detecting probe was synthesized by sequentially coupling NIR dye and CQRPPR into a polyethylene glycol (PEG) polymer. The ability of the probe to bind apoptotic cells was first tested *in vitro* with the use of apoptotic chondrocytes. The effectiveness of the probe to detect chondrocyte apoptosis in injured mouse xiphoid explants was further demonstrated. Finally, this probe was tested for its ability to detect chondrocyte apoptosis *in vivo* using our previously described xiphoid injury mouse model^25^.

## Results

**Figure 1:**
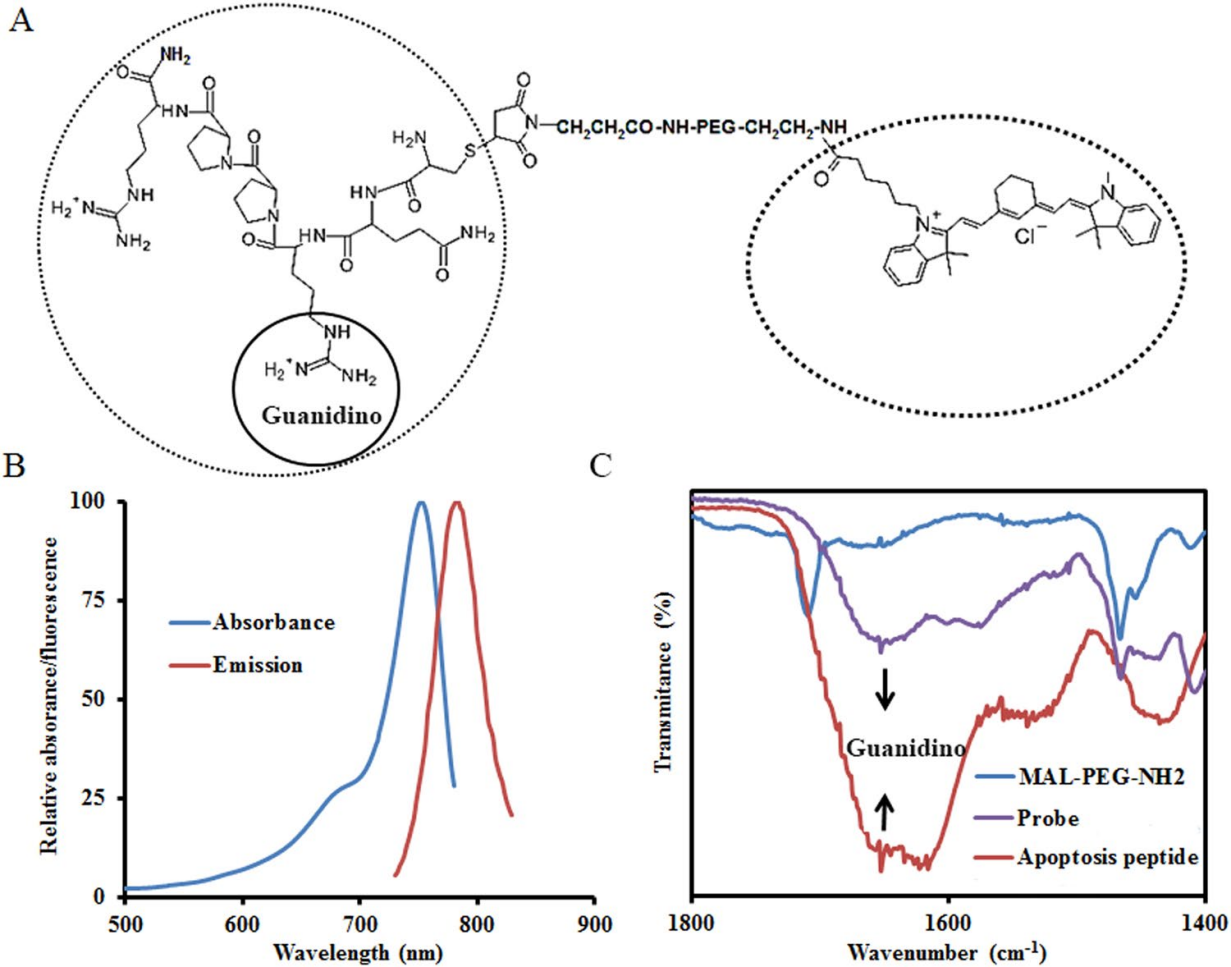
The preparation and characterization of the apoptotic cell-detecting probe. (**A**) Schematic illustration of the probe structure. (**B**) The optical properties of the probe. (**C**) Fourier transform infrared spectra of the peptide, the polyethylene polymer, and the probe, respectively.

The apoptotic cell-detecting probe was prepared, and its structure is schematically shown in Fig. 1A. PEG was introduced into the probe to improve biocompatibility of the probe and water solubility, as well as to extend its circulation time in the body, as described in previous studies^41, 42^. Optical measurement of the probe showed that the probe had a maximum absorbance peak at 755 nm and a maximum emission peak at 787 nm (Fig. 1B). These results showed that the NIR dye was successfully coupled with the probe. Fourier transform infrared spectra (FT-IR) were created with the use of a Nicolet 6700 spectrometer to determine the chemical structure of the probe. A broad band from 1,620 to 1,660 cm^−1^ for both the peptide and the probe was identified, and the band is typically assigned to the guanidino group of the peptide (Fig. 1C)^43, 44^. The FTIR results indicated that the apoptotic cell-binding peptide had been conjugated successfully with the probe.

**Figure 2:**
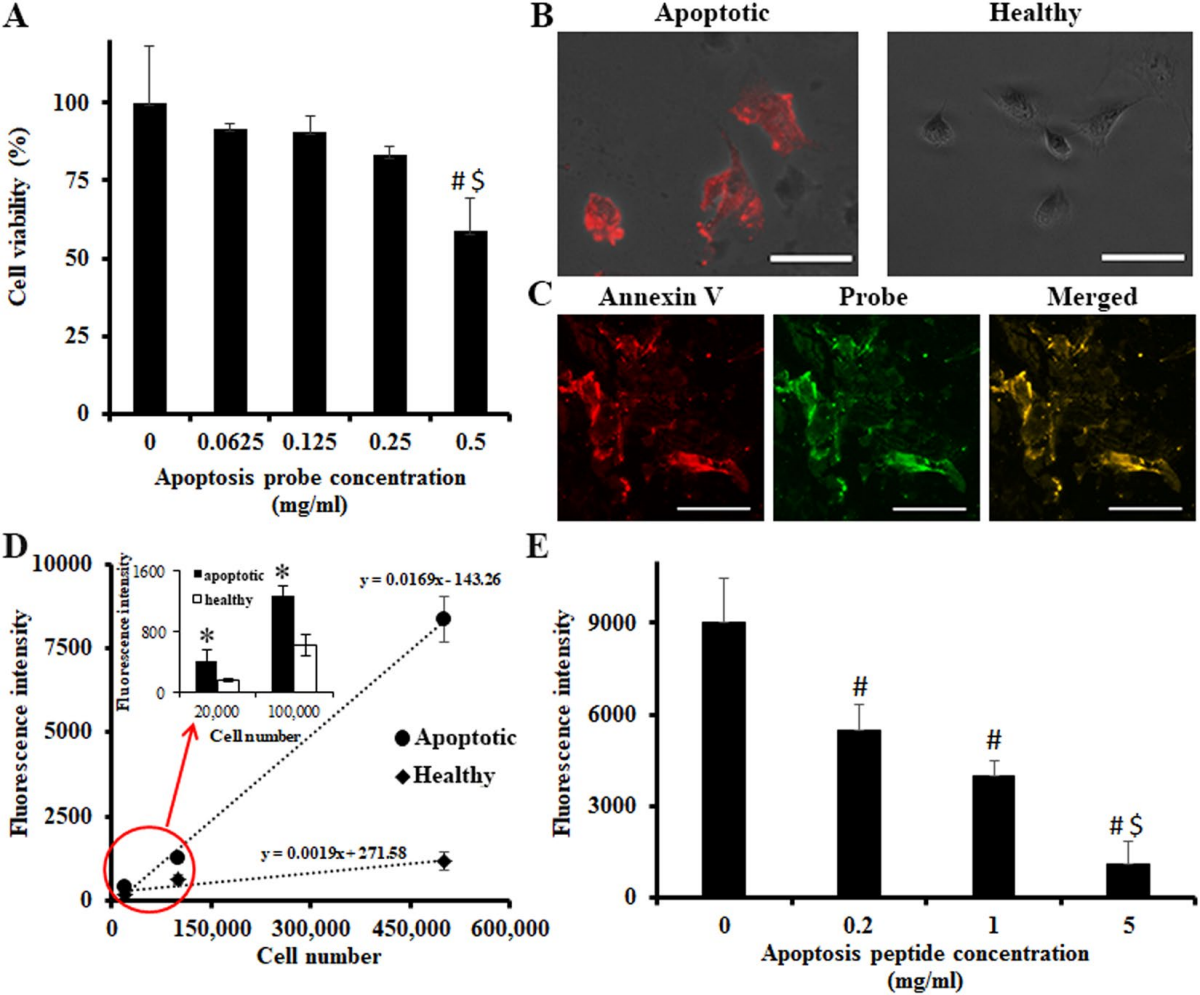
*In vitro* study to assess the cytotoxicity of the probe to cells and to evaluate the binding ability of the probe to apoptotic chondrocytes. (**A**) Cytotoxicity study of the probe on the cells using an MTT assay. (n = 5, # and $ indicate p < 0.05 versus 0 mg/ml and 0.0625 mg/ml, respectively. ANOVA p < 0.05). (**B**) Annexin V staining: apoptotic cells (left) and healthy cells (right). Scale bar: 50 μm. (**C**) Co-staining of SNP-treated apoptotic chondrocyte with Annex V (left), the probe (middle) and their superimposed image (right). Scale bar: 100 μm. (**D**) The effect of the apoptotic bovine chondrocyte number and healthy bovine chondrocyte number on the fluorescence intensity. Cell apoptosis induced by SNP (1 mM) for 24 hours and probe incubation (0.05 mg/mL) for 30 minutes. (n = 5, Apoptotic cells: Pearson coefficient = 0.9984 Healthy cell: Pearson coefficient = 0.9506). (**E**) The effect of free peptide in various concentrations on reducing the binding of apoptosis probes to apoptotic cells (500,000 cells per well). Unconjugated peptides were added to block Histone H1 on apoptotic cells prior to addition of apoptosis probe (0.05 mg/mL, 30 minutes incubation) to test probes’ specificity to apoptosis cells (n = 5, ANOVA p < 0.05 among groups, # and $ indicate p < 0.05 versus 0 mg/ml and 0.2 mg/ml, respectively). All the experiments were confirmed statistically using ANOVA with Tukey-Kramer test or Pearson coefficient. All the statistical data were presented as mean ± standard deviation.

Next, the cytotoxicity of the probe was tested using bovine chondrocytes and an MTT assay (Fig. 2A). The results of this experiment showed that the probe did not induce a statistically significant cytotoxicity to chondrocytes under the concentration of 0.25 mg/mL. On the basis of these result, subsequent studies were carried out using a probe concentration of 0.05 mg/mL. The binding ability of the probe to apoptotic cells was further evaluated using apoptotic bovine chondrocytes. Bovine chondrocytes were first treated with sodium nitroprusside (SNP) to induce apoptosis. Apoptotic chondrocytes were confirmed using Annexin V Fluorescence 594 (Fig. 2B). Annexin V staining (red color) was found only in SNP-treated cells. Next, to confirm the specificity of the probe, SNP-treated apoptotic chondrocytes were co-stained with Annexin V and the probe. Co-localization of two different markers was observed under a fluorescence microscope (Fig. 2C). Merged picture (yellow) of Annexin V (red) and the probe (green) demonstrated the probe has great specificity to apoptotic cells. Furthermore, apoptosis-associated fluorescence intensity of the probe was determined *in vitro* (Fig. 2D). One can observe that the fluorescence intensity increases with the increase in SNP-treated chondrocyte number. At the same time, increasing number of healthy chondrocytes only contributed a slight increase in fluorescence intensity. There is a very high positive correlation between the number of apoptotic chondrocytes and the fluorescence intensities (Pearson correlation coefficient = 0.998 for apoptotic cells and Pearson correlation coefficient = 0.962 for healthy cells) (Fig. 2D). Also, there is a significant difference between the fluorescent intensities of apoptotic and healthy cells, when comparing cell numbers ranging from 20000 to 500000 (Student’s t-test, p < 0.05) (Fig. 2D Insert). These results support that the probe can be used to detect the numbers of apoptotic cells as few as 20000 cells *in vitro*. To further confirm the role of the peptide portion of the probe in the targeting of apoptotic cells, a competition binding test was conducted in which peptides, competing for cell targeting, with various concentrations were introduced to the culture media before the addition of probes (Fig. 2E). As expected, the fluorescence intensity was markedly decreased in the presence of increasing concentrations of peptides. There was an approximate 70% reduction in fluorescence intensity when the peptide (5 mg/mL) was added in the competition tests (ANOVA with Tukey-Kramer’s test, p < 0.05, # and $ indicates p < 0.05 versus 0 mg/ml and p < 0.05 versus 0.2 mg/ml, respectively). The results suggested that probe-to-cell interactions were mediated by the ApoPep-1 peptide: cell membrane interaction rather than by cell phagocytosis of probe. Overall, the *in vitro* studies demonstrated that the probe had a high affinity to apoptotic cells and that it could therefore be used to quantify the amount of apoptotic cells both *ex vivo* and *in vivo*.

**Figure 3:**
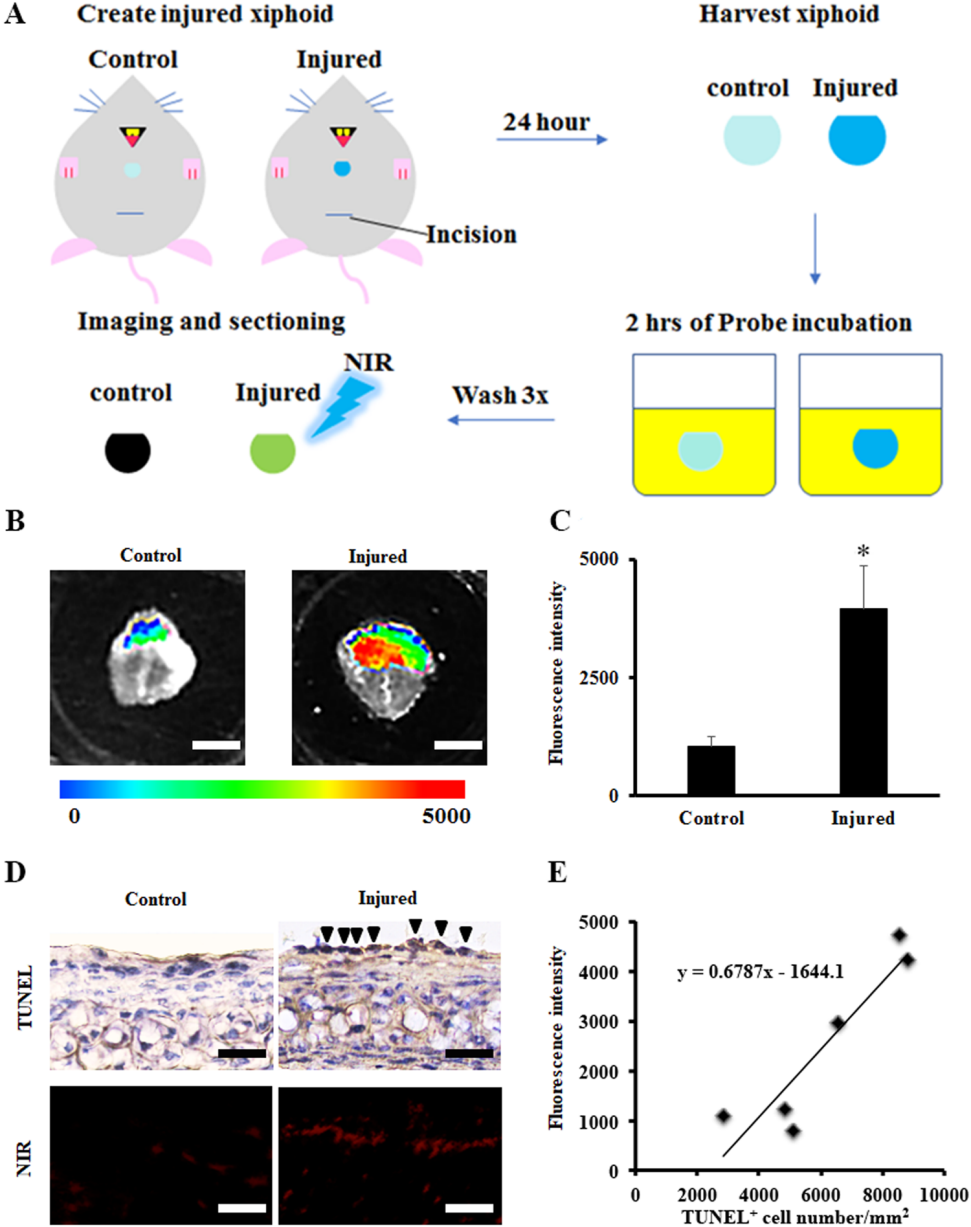
*Ex vivo* study to assess the targeting ability of the probe to apoptotic chondrocytes of injured xiphoid. (**A**) A schematic illustration of *ex vivo* injured xiphoid model. (**B**) *Ex vivo* imaging of injured xiphoid tissue. Probe incubation (0.05 mg/mL) for 2 hours. Scale bar: 2 mm. (**C**) Quantification analysis (n = 5, Student’s t-test, *p < 0.05). (**D**) TUNEL staining of injured cartilage explants and control tissue, and near-infrared fluorescence images of injured and healthy (control) xiphoid tissues sections (Scale bar: 100 μm. Arrow heads indicate TUNEL positive cells). (**E**) Correlation between fluorescence intensity and TUNEL positive chondrocyte number per 1 mm^2^. Pearson correlation test was performed (Pearson coefficient = 0.9193). All the statistical data were presented as mean ± standard deviation.

We first assessed whether this probe could be used to detect injured cartilage *ex vivo* with the use of isolated injured xiphoid tissue and controls as drawn schematically (Fig. 3A). We found that after incubating the tissues with the probe at 37 °C for 2 hours, there was significantly greater florescence intensity from the areas of the xiphoid where the clamp had been applied (Fig. 3B). Quantification analysis showed a four-fold greater fluorescence intensity in the injured xiphoid as compared to the control tissue (Student’s t-test, *p < 0.05) (Fig. 3C). As demonstrated in a previous study, TUNEL staining revealed that the mechanical compression indeed triggered chondrocyte apoptosis (Fig. 3D)^23^. Using a fluorescence microscope, we found that probe accumulation on the surface of the injured tissues was much higher than control tissues (Fig. 3D). Correlation analysis showed a very high positive relationship between fluorescence intensity and apoptotic (TUNEL+) chondrocyte number (Pearson correlation coefficient: 0.919) (Fig. 3E). These results support the notion that this probe could be used as an indicator to identify injured cartilage *ex vivo* early in the degeneration process.

**Figure 4:**
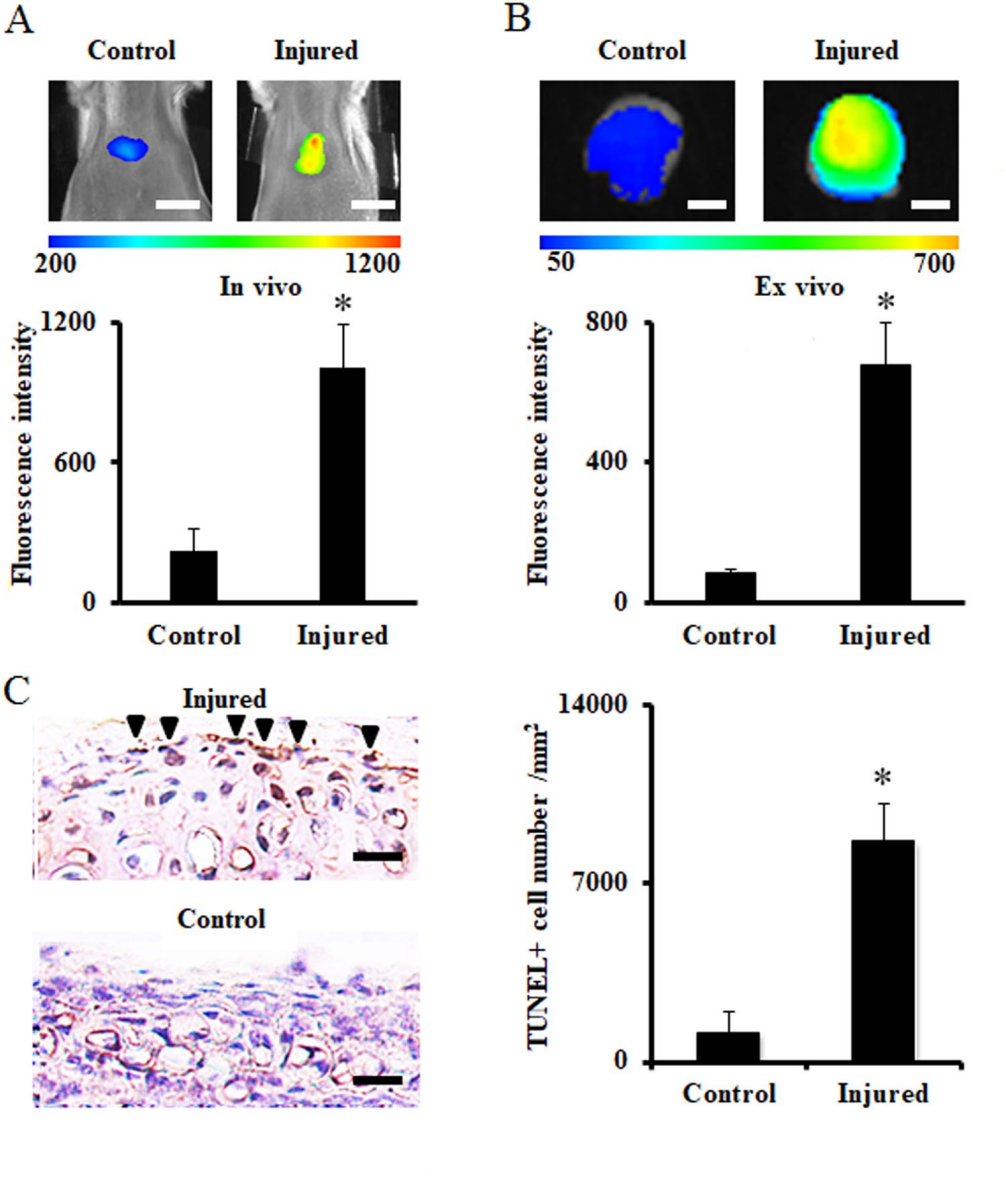
*In vivo* imaging of the xiphoid injury mouse model. (**A**) The merged fluorescence signal with a white-light image captured on the fifth day after xiphoid injury. Probe injection was performed at concentration of 0.05 mg/mL 24 hours before imaging (top) and the quantification analysis of signal intensity (bottom). Scale bar: 1.0 cm. (**B**) *Ex vivo* imaging of xiphoid tissue (top) and the quantification analysis (bottom). *Ex vivo* xiphoid samples were isolated from *in vivo* mouse model after imaging. Scale bar: 2.0 mm. (**C**) TUNEL staining of xiphoid (left) and the quantification analysis (right) (Arrow heads indicate TUNEL positive cells). Number of TUNEL positive cells was counted and normalized to 1 mm^2^. All the data were calibrated with Student’s t-test (n = 5, *p < 0.05). Scale bar: 100 μm. All the statistical data were presented as mean ± standard deviation.

Next, we used a mouse model to investigate whether the probe was able to detect apoptotic chondrocytes using a mechanically-triggered injured xiphoid model *in vivo*. Four days after the xiphoid was injured, the apoptosis probe (100 μL at 0.05 mg/mL) was administered intraperitoneally. *In vivo* images were captured on the fifth day after xiphoid injury (Fig. 4A). We found that the probe accumulated considerably more in injured xiphoid tissues than in control specimens. Fluorescence intensity in the injured xiphoid was approximately five times higher than that of the control group. To further verify that the signal came from the xiphoid, mice were euthanized, and their xiphoids were isolated for additional *ex vivo* imaging. Although the signal intensity for *ex vivo* imaging was a little lower than that of the *in vivo* imaging, the signal-to-noise ratio was profoundly higher (8×, Student’s t-test, *p < 0.05) (Fig. 4B) as compared with that of the *in vivo* imaging (5×, Student’s t-test, *p < 0.05) (Fig. 4A). TUNEL staining was performed to quantify the numbers of apoptotic chondrocytes and TUNEL-positive cell numbers were normalized to 1 mm^2^. Approximately 8 times more TUNEL-positive cells (apoptotic cells) were observed in the injured xiphoid tissue as compared with the control tissue (Fig. 4C). These experiments confirmed that the apoptosis probe could be used to detect injured cartilage undergoing injury-related degeneration in an otherwise intact animal.

**Figure 5:**
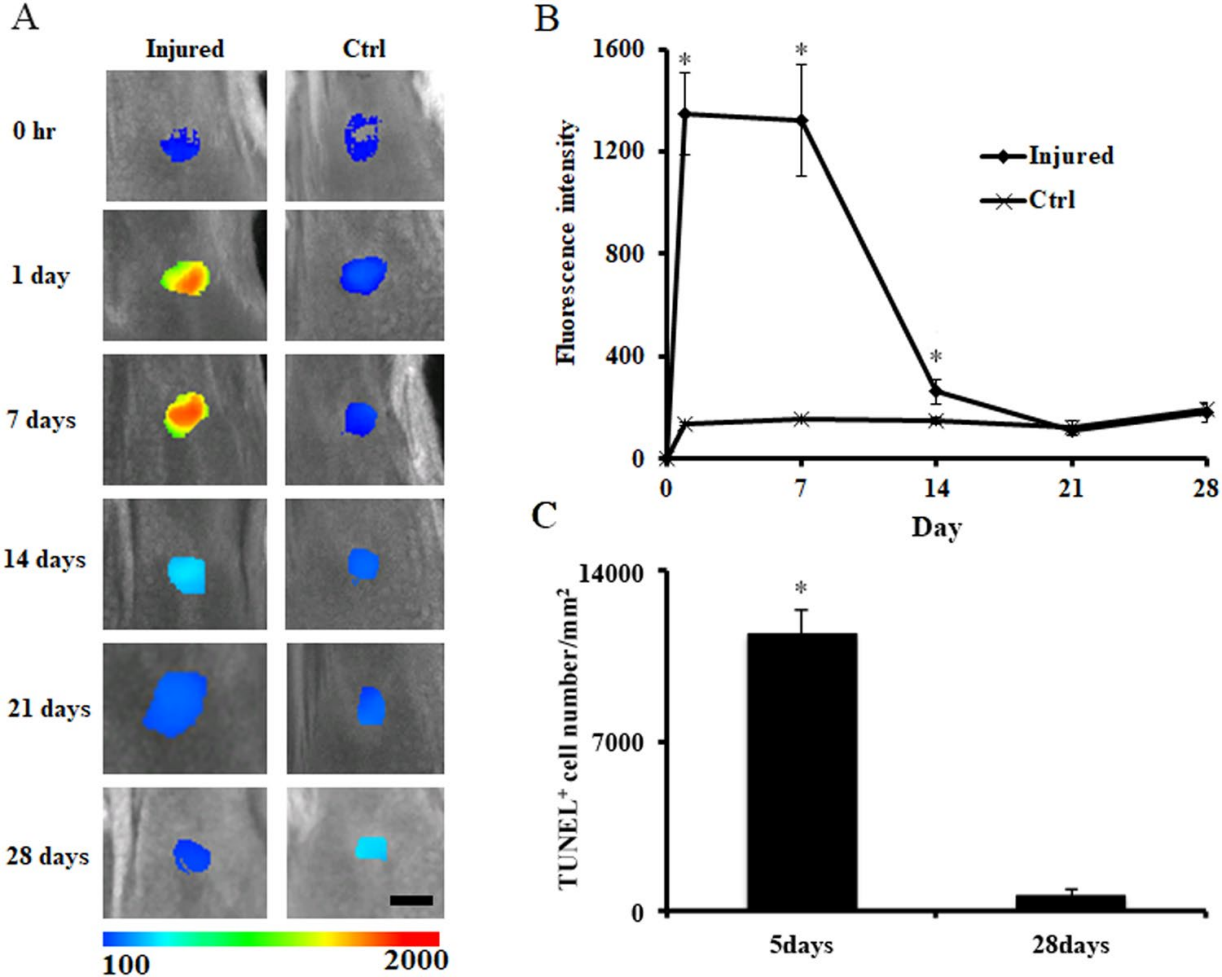
Dynamic monitoring of chondrocyte apoptosis in the xiphoid injury mouse model. (**A**) *In vivo* imaging over time. Sequential images were captured from the same mouse. Scale bar: 4.0 mm. (**B**) Fluorescence intensity changes at different time points after probe injection (n = 5, Student’s t-test, * indicates p < 0.05 versus control). (**C**) Quantitative analysis of apoptotic xiphoid cells. Xiphoid injury was created at Day 0. After the NIR images were taken at the specific time points (Day 5 or Day 28), the animals were sacrificed and xiphoid tissues were isolated for histological analyses. TUNEL positive cells were counted and then normalized to cell number per mm^2^. (n = 5, Student’s t-test, *p < 0.05). All the statistical data were presented as mean ± standard deviation.

We examined the potential use of apoptotic probes to monitor recovery after xiphoid injury. The probes were administered 24 hours before the *in vivo* imaging detection was performed. As predicted by our early observations, strong fluorescence appeared only at the injured xiphoid sites but not at the control sites (Fig. 5A). Interestingly, strong fluorescence signals were able to be observed at the injured xiphoid sites between Day 1 and 7 after injury (Student’s t-test, * indicates p < 0.05 versus control) (Fig. 5A and B). The reduced fluorescence intensity that occurred after Day 14 suggested the reduction of the apoptotic cartilage cells and the recovery of the cartilage tissues; these findings concur with the histological evaluation results (TUNEL assay, Student’s t-test, *p < 0.05) (Fig. 5C).

## Discussion

The molecular design of the NIR imaging probe was very critical for *in vivo* imaging. In this investigation, a histone H1-targeting peptide—CQRPPR—was chosen as a targeting ligand for apoptotic cells. Previous studies have demonstrated that the H1-targeting peptide can bind H1 that is exposed on the surface of apoptotic cells^39, 40^. Based on the peptide, several probes had been designed for the diagnosis of tumor apoptosis, myocardial cell death, and cell apoptosis associated with ischemia^45, 46^. To fabricate apoptosis probes for injured cartilage detection, PEG polymer was included as a carrier to reduce nonspecific binding and to enhance bioavailability and clearance. An NIR dye, Sulfo-Cyanine7, was selected for its deeper penetration depth and its lower rate of absorbance into the gaps of tissues with improved probe sensitivity^16^.

*In vitro* binding tests of apoptotic cells demonstrated that the probe had strong binding ability to apoptotic chondrocytes. Competition experiments further revealed the critical role of the targeting peptide for the probe to bind apoptotic cells. These results suggest that the binding of the probe to apoptotic cells occurred through their interactions with histone H1 on the surfaces of apoptotic chondrocytes, which concurred with the results of previous studies^45–47^. Our xiphoid injury model was used to investigate whether apoptotic chondrocytes could be detected using the probe^25^. *Ex vivo* imaging experiments confirmed that the probe could be used to identify and assess the extent of chondrocyte apoptosis induced by mechanical compression. There is also a positive relationship between the extent of fluorescence intensity and the number of apoptotic chondrocytes. These results suggest that the probe could be used not only to detect chondrocyte apoptosis but also to estimate the extent of the apoptotic chondrocytes.

Finally, via the intraperitoneal administration of the apoptosis probe, *in vivo* imaging was carried out to evaluate the effectiveness of the probe for the detection of chondrocyte apoptosis *in vivo*. Our results have shown that there was significantly more probe accumulation at the injury sites as compared to control sites. By continuously monitoring the probe’s affinity for xiphoid tissue at different time points, we found that apoptotic cells were most prominent between day 1 and day 7 after injury. The probe accumulation at the injured xiphoid sites was drastically reduced after day 14, which suggests loss of apoptotic chondrocytes from the xiphoid tissue after clamp-induced mechanical injury.

Our results show great promise regarding the use of the probe for the *in vivo* identification of chondrocyte apoptosis. However, it should be noted that xiphoid injury is not completely identical to articular cartilage injury that would occur in a joint such as the knee. Further experiments will need to be performed using an animal model of articular cartilage injury to investigate whether the probe can detect apoptotic chondrocytes in recently injured articular cartilage. Furthermore, the limited tissue penetration depth of the NIR signal (could be up to 10 cm depending on the tissue types)^15^ may hinder the probe’s application in clinical practice. To overcome such deficiencies and to directly visualize the injured cartilage, the probe may be labeled with radionucleotide and specific contrast agents for other imaging modalities (e.g. positron emission tomography, and magnetic resonance imaging)^42, 45, 48^.

In conclusion, an apoptotic cell-detecting optical imaging probe based on a histone H1-binding peptide has been developed to target apoptotic cells. The probe has a high affinity for apoptotic chondrocytes *in vitro* and for the mechanical force–associated cartilage explants *ex vivo*. With the use of a xiphoid injury model in mice, we found that the probe was able to detect apoptotic cells associated with injured xiphoid *in vivo*. These results demonstrate that the probe may provide a rapid noninvasive imaging technique for the diagnosis of cartilage apoptosis associated with post-traumatic OA.

## Methods

An apoptotic cell-binding peptide (CQRPPR) (United BioSystems Inc., Herndon, VA); a sulfo-cyanine7 N-hydroxysuccinimide ester (Cy7®, Lumiprobe Corp., Hallandale Beach, FL); and a heterobifunctional maleimide polyethylene glycol amine (Mw:3.5k, Mal-PEG-NH2) (JenKem Technology USA, Plano, TX) were purchased. All other chemicals used in this investigation were purchased from Sigma-Aldrich Corporation (St Louis, MO).

### Preparation of the apoptotic cell-detecting probe

The apoptotic cell-detecting probe was prepared by the conjugation of CQRPPR and NIR dye onto both ends of the polyethylene glycol. Specifically, dye and Mal-PEG-NH2 were dissolved in phosphate-buffered saline (PBS; pH 8.5) at a 2:1 molar ratio and incubated at room temperature overnight. After removing the unconjugated dye by dialysis against deionized water, dye-labeling PEG was collected via freeze-drying. The dye-labeling PEG and peptide were dissolved in PBS (pH 7.2) at a 1:20 molar ratio and incubated overnight at 4 °C. After dialysis against deionized water, the probe was lyophilized and stored at 4 °C for further use. Absorbance and fluorescence spectra of the probe were measured with the use of an ultraviolet-visible spectrophotometer (Lambda 19 Spectrometer, Perkin Elmer, MA) and an Infinite M200 microplate reader (Tecan, San Jose, CA), respectively. The probe was further analyzed with the use of a Nicolet 6700 FT-IR spectrometer (Thermo Nicolet Corp., Madison, WI). To stain cells for microscope observation, some apoptosis probes were also prepared with Cy5® dye (sulfocyanine7 N-hydroxysuccinimide ester).

### Cytotoxicity of the probe

The cytotoxicity of the probe to cells was determined via MTT assay as described previously^49, 50^. Bovine chondrocytes were isolated from the patella-femoral grooves of calves using previously established protocols^51^. Briefly, calf knee joints were harvested from a local abattoir less than 36 hours after slaughter. Articular cartilage for *in vitro* study was collected from the patella-femoral grooves of only one donor, minced to approximately 1 mm^3^ pieces, and digested with 2 mg/mL collagenase type II (Worthington Biochemical Corp., Lakewood, NJ) in Dulbecco’s Modified Eagle’s Medium with 1% penicillin–streptomycin–fungizone (Life Technologies Corp., Carlsbad, CA) at 37 °C overnight. The chondrocyte solution was filtered through a 70 μm cell strainer, centrifuged, and then cultured in Dulbecco’s Modified Eagle’s Medium (DMEM). Six thousand cells were plated into each well of a 96-well plate and incubated overnight (37 °C), and then probes at various concentrations were added to the plate. After 24 hours of probe incubation (0 to 0.5 mg/ml), the MTT assay was carried out with the use of a SpectraMax 340 spectrophotometer (Molecular Devices, Sunnyvale, CA).

### Fluorescence microscopy

To stimulate chondrocyte apoptosis, sodium nitroprusside (SNP) was used as described previously^52^. Briefly, bovine chondrocytes were incubated with SNP (1 mM) in DMEM for 24 hours. To determine SNP-induced apoptosis, these SNP-treated chondrocytes were stained with Annexin V Fluorescence 594 (Molecular probes, Eugene, OR) following manufacturer’s protocol. Further, the SNP-treated apoptotic chondrocytes were co-stained with Annexin V and the Cy5-labeling probe (0.05 mg/mL, 30 minutes). After washing twice with PBS, cells were observed on a Leica DMi8 Fluorescence microscope (Leica, Wetzlar, Germany). Cy3 and Cy5 channels were used in detecting Annexin V Fluorescence 594 and Cy5-probe, respectively. All the microscopic images were captured under 200x magnification and analyzed with ImageJ.

### *In vitro* assessment of the probe’s affinity for apoptotic cells

To assess the ability of the probe to detect apoptotic bovine chondrocytes *in vitro*, the SNP-treated apoptotic chondrocytes and untreated chondrocytes (from 20,000 to 500,000 cells per well) as described above were incubated with the probe (10 μL at 0.05 mg/mL) at 37°C in the 96-well plate. After 30 minutes of probe incubation, each well was washed three times with PBS (pH 7.4) to remove the unbound probes. Apoptosis-associated fluorescence intensities were then determined via the Infinite M200 microplate reader at an excitation wavelength of 760 nm and an emission wavelength of 830 nm. For blocking tests, various concentrations of CQRPPR peptides (without NIR dye conjugation) were added to the chondrocyte-seeded wells (500,000 cells per well) and incubated for 10 minutes before the addition of the probe (10 μL at 0.05 mg/mL). After 30-minute incubation with the probe, the fluorescence intensity was then measured after excessive probes were removed with 3-time PBS washing.

### *Ex vivo* and *in vivo* evaluation of the apoptotic probe using the xiphoid injury model

To investigate whether the probe could detect apoptotic chondrocytes in injured cartilage, we employed a previously validated model that made use of mouse xiphoid cartilage explants^25^. All animal experiments were approved by the University of Texas at Arlington Animal Care and Use Committee (IACUC), in accordance with the Animal Welfare Act, and consistent with the Guide for the Care and Use of Laboratory Animals. In addition, all research involving animals must comply with the Public Health Service “Policy on Humane Care and Use of Laboratory Animals”. The animal procedure is summarized below. Briefly, Balb/c mice (6 to 8 weeks old) were obtained (Taconic Farms, Inc., Germantown, NY) for each study. To trigger xiphoid chondrocyte apoptosis *in vivo*, the mice were anesthetized, and an incision (1 cm) was created in the midline of the upper abdomen to expose xiphoid. The xiphoid was then injured by clamping for 2 minutes with a modified Kelly hemostatic clamp (tip, 9.2 mm long × 4.6 mm wide; item no. BH443R; Aesculap, Inc., Center Valley, PA), as previously described^25^. For control purposes, identical incisions were made on additional mice without clamping of the xiphoid. For *ex vivo* analysis, several of the animals were euthanized, and the xiphoid tissue was harvested. The injured and uninjured (control) xiphoid tissues were incubated with probes (0.05 mg/mL) for 2 hours. The injured cartilage was washed three times with PBS (pH 7.4), and then *ex vivo* imaging was performed with a Kodak *In Vivo* FX Pro system at an excitation wavelength of 760 nm and an emission wavelength of 830 nm. For *in vivo* evaluation, mice were first divided into injured and control groups. *In vivo* injured xiphoid model was created by clamping the xiphoid structure for 2 minutes, and no damage was made in control groups. On the fourth day after injury, the probe (100 μL at 0.05 mg/mL) was injected intraperitoneally into two groups of mice. 24 hours later, *in vivo* images were taken and xiphoid cartilages were obtained from mice. To confirm probe accumulation, *ex vivo* images of xiphoid were also captured. For long-term observation, xiphoid injury in mice was created and then images were immediately taken at Day 0. For the next few weeks, the probe (100 μL injection at 0.05 mg/mL) was administered every time 24 hours before each image was taken with a Kodak *In Vivo* FX Pro system.

### Immunohistochemistry and histology

To directly assess the xiphoid tissue for evidence of apoptosis, injured and uninjured specimens were embedded in the optical cutting temperature compound. The xiphoid sections (8 μm in thickness) were made in a rostrocaudal manner. TUNEL staining was carried out to determine the presence of apoptotic cells. Apoptotic cells were then labeled in accordance with the manufacturer’s instructions (TUNEL Apoptosis Detection Kit; GenScript, Piscataway, NJ). In brief, sections were first fixed in 4% paraformaldehyde (in phosphate buffered saline, PBS), and then sections were incubated with TUNEL Reaction Mixture following blocking process (3% H_2_O_2_) and permeabilization step (0.1% Triton X-100 in 0.1% sodium citrate). After reacting with anti-fluorescein Antibody Solution, sections were incubated in DAB substrate solution. Between all the steps, sections were washed with PBS for several times. TUNEL Apoptosis Detection Kit contains most of the reagents except fixation buffer, blocking solution, and permeabilization solution. Hematoxylin was used for counterstaining. The images were taken under a microscope (Leica). Using ImageJ, TUNEL positive cells were counted per field of view (200x magnification) under the same area (1 mm^2^) from different sample sections as cell density. All quantification data for histological analysis were normalized to cell number per mm^2^.

### Statistical analysis

ANOVA with Tukey Kramer’s test was used to perform the statistical analysis for all the data obtained from the different treatment groups. All the *in vitro*, *ex vivo* and *in vivo* experiments were repeated 5 times. Differences were designated as statistically significant when P ≤ 0.05 (Student’s t-test). Pearson correlation coefficient was also conducted to reflect the relationship between fluorescence intensities and apoptotic cell numbers *in vivo*. All the statistical results were presented following the format of mean ± standard deviation. ANOVA with Tukey-Kramer test was analyzed in Fig. 2A and Fig. 2E. Pearson coefficients for Fig. 2D and Fig. 3E were calibrated. Student’s t-test was performed in Fig. 2D Insert, Fig. 3C, Fig. 4, Fig. 5B and Fig. 5C.
